# Long-term Outcomes after Salvage Stereotactic Radiosurgery (SRS) following In-Field Failure of Initial SRS for Brain Metastases

**DOI:** 10.3389/fonc.2017.00279

**Published:** 2017-11-23

**Authors:** Nitesh Rana, Praveen Pendyala, Ryan K. Cleary, Guozhen Luo, Zhiguo Zhao, Lola B. Chambless, Anthony J. Cmelak, Albert Attia, Mark J. Stavas

**Affiliations:** ^1^Department of Radiation Oncology, Vanderbilt University Medical Center, Nashville, TN, United States; ^2^Department of Biostatistics, Vanderbilt University Medical Center, Nashville, TN, United States; ^3^Department of Neurological Surgery, Vanderbilt University Medical Center, Nashville, TN, United States

**Keywords:** brain metastases, stereotactic radiosurgery, radionecrosis, reirradiation, repeat SRS

## Abstract

**Purpose:**

The optimal treatment strategy following local recurrence after stereotactic radiosurgery (SRS) remains unclear. While upfront SRS has been extensively studied, few reports focus on outcomes after retreatment. Here, we report the results following a second course of SRS for local recurrence of brain metastases previously treated with SRS.

**Methods:**

Using institutional database, patients who received salvage SRS (SRS2) following in-field failure of initial SRS (SRS1) for brain metastases were identified. Radionecrosis and local failure were defined radiographically by MRI following SRS2. The primary endpoint was defined as the time from SRS2 to the date of all-cause death or last follow-up [overall survival (OS)]. The secondary endpoints included local failure-free survival (LFFS) and radionecrosis-free survival, defined as the time from SRS2 to the date of local failure or radionecrosis, or last follow-up, respectively.

**Results:**

Twenty-eight patients with 32 brain metastases were evaluated between years 2004 and 2015. The median interval between SRS1 and SRS2 was 9.7 months. Median OS was 22.0 months. Median LFFS time after SRS2 was 13.6 months. The overall local control rate following SRS2 was 84.4%. The 1- and 2-year local control rates are 88.3% (95% CI, 76.7–100%) and 80.3% (95% CI, 63.5–100%), respectively. The overall rate of radionecrosis following SRS2 was 18.8%. On univariate analysis, higher prescribed isodose line (*p* = 0.033) and higher gross tumor volume (*p* = 0.015) at SRS1 were associated with radionecrosis. Although not statistically significant, there was a trend toward lower risk of radionecrosis with interval surgical resection, fractionated SRS, lower total EQD2 (<50 Gy), and lack of concurrent systemic therapy at SRS2.

**Conclusion:**

In select patients, repeat LINAC-based SRS following recurrence remains a reasonable option leading to long-term survival and local control. Radionecrosis approaches 20% for high risk individuals and parallels historic values.

## Introduction

Brain metastases account for over half of brain tumors in adults (1–3). With increasing utilization of magnetic resonance imaging and improved systemic therapies, the incidence of brain metastases is increasing (2, 3). In addition, the median survival for patients with brain metastasis has improved over time due to earlier detection, better brain-directed therapies, and more effective systemic therapies (4, 5). Simultaneously, therapeutic strategies are shifting away from whole brain radiation therapy (WBRT) because of growing concerns about neurocognitive toxicity (6). Recent data suggest that stereotactic radiosurgery (SRS) alone is the preferred modality for individuals with one to three brain metastases (7), with some studies demonstrating favorable outcomes in individuals with greater than four brain metastases, especially younger individuals with limited extracranial disease (8, 9).

Stereotactic radiosurgery is an effective modality for treating brain metastases, delivered as either singular treatment or as adjuvant treatment after surgical resection, with 1-year local control rates approaching 90% (10, 11) and minimal toxicity (6, 12). However, given that some patients with brain metastases are living beyond 1 year with the advent of novel targeted therapies (13, 14), the management of local recurrence needs to be studied further. Data regarding the optimal treatment approach for these individuals are incredibly sparse.

Currently, treatment options include WBRT, surgical resection, repeat SRS, or best supportive care. The aim of this study was to analyze patient and disease outcomes, including safety and efficacy, using salvage repeat LINAC-based SRS following in-field failure after initial SRS for brain metastases.

## Materials and Methods

### Data Acquisition

Following institutional board approval, we performed a single-institutional retrospective study. Patient data were obtained from a prospectively maintained database of patients treated with SRS for brain metastases. Patients who received a repeat course of SRS (SRS2) to an isocenter that was previously treated with SRS (SRS1) were identified. Composite treatment plans were created for each course of repeat SRS using Brainlab (Munich, Germany) iPlan software. Isodose lines, hotspots, dose and volume histograms, cumulative dose, and EQD2 were analyzed. Patients with less than 50% overlap of the prescribed dose between SRS1 and SRS2 were excluded for analysis. Patients who had surgical resection in between SRS1 and SRS2 were included. All lesions were treated with the Novalis TX equipped with Brainlab ExacTrac Localization Systemic and iPlan Treatment Planning Software.

Radionecrosis and local failure were defined radiographically by MRI following SRS2. Electronic medical records were reviewed to determine patient characteristics including age, sex, histological findings, extracranial disease status, Karnofsky Performance Scale (KPS) score, Diagnosis-Specific Graded Prognostic Assessment (DS-GPA), prior WBRT, and prior surgery. Outcomes including local control, toxicity, radiation necrosis, and death were also determined *via* electronic medical records.

### Radiosurgery Technique

The gross tumor volume (GTV) for each course of SRS was determined from a stealth (1 mm slices) T1 post contrast-enhanced MRI of the brain. The MRI images were digitally registered to a head CT obtained during treatment simulation following in-house SRS scanning protocol. Immobilization for each treatment was achieved using a non-rigid mask. Treatment planning was performed using Brainlab iPlan system. For all the SRS deliveries, ExacTrac image-guided system with 6 degree-of-freedom couch was used for localization. Dose and fractionation were determined by the treating physician based on volume, size, histology, prior resection, and previous radiation history.

### Response Assessment

Patients were followed with serial MRIs every 2–3 months after each course of SRS. Local failure was defined as serial increase in enhancement on MRI, either pathologically proven or continued serial increase in enhancement not responsive to steroids. Date of first MRI suggestive of local failure was used for calculation. Radionecrosis was defined as enhancement on serial MRI, either pathologically proven or serial enhancement ultimately stabilized with use of steroids. Other imaging modalities, such as MR PET and perfusion-weighted MRI, were also taken into account. Date of first MRI suggestive of radionecrosis was used for calculation.

### Statistics

Patients’ and lesions’ characteristics were summarized using the median with the 25th and 75th percentiles (interquartile range) for continuous variables. For categorical variables, frequency and percentages were shown. The primary endpoint was defined as the time from SRS2 to the date of all-cause death or last follow-up [overall survival (OS)]. The secondary endpoints included local failure-free survival (LFFS) and radionecrosis-free survival (RFS), defined as the time from SRS2 to the date of local failure or radionecrosis, or last follow-up, respectively. The Kaplan–Meier method, Log-rank test, and Cox proportional hazard models were used in univariate survival analyses when appropriate to investigate the associations between the endpoints and patients/lesions characteristics. For visualization purposes, prescribed isodose line and GTV volume at SRS1 were dichotomized by the medians in the Kaplan–Meier plots for RFS. All statistical inferences were assessed at a two-sided 5% significant level, and all summary statistics, graphics, and survival models were generated using R version 3.3 statistical software (15).

## Results

### Patients and Treatment Characteristics

Between 2004 and 2015, 11 females and 17 males received a second course of SRS to 32 brain metastases initially treated with SRS. Patient and lesion characteristics are summarized in Table 1. Fourteen of the lesions were melanoma, five were renal cell carcinoma, five were breast carcinoma, four were non-small cell lung cancer, two were small cell lung cancer, one was sarcoma, and one was testicular cancer. Eleven lesions were located in the frontal lobe, seven lesions in the parietal lobe, five lesions in the temporal lobe, five lesions in the cerebellum, and four lesions in the occipital lobe. Ten patients (11 brain metastases) received WBRT prior to SRS1, with a median interval of 10.7 months (range 2.9–69.6 months) in between WBRT and SRS1. Median age at SRS1 was 58 years (range, 32–77 years).

**Table 1:** Baseline characteristics of patients and of lesions.

Patient characteristics	Value (%)	Lesion characteristics	Value (%)
Number of patients	28	Number of lesions	32
Sex		Histology	
Male	17 (61)	Melanoma	14 (44)
Female	11 (31)	RCC	5 (16)
Median age at SRS1 (years)	58	Breast	5 (16)
Median age at SRS2 (years)	60	NSCLC	4 (12)
Histology		SCLC	2 (6)
Melanoma	11 (39)	Sarcoma	1 (3)
RCC	5 (18)	Testicular	1 (3)
Breast	5 (18)	Location	
NSCLC	3 (11)	Frontal	11 (34)
SCLC	2 (7)	Parietal	7 (22)
Sarcoma	1 (4)	Temporal	5 (16)
Testicular	1 (4)	Occipital	4 (12)
Prior WBRT	8 (33)	Cerebellar	5 (16)
KPS score at SRS2		SRS1	
80–100	23 (82)	Single fraction	30 (94)
70-	5 (18)	Fractionated	2 (6)
		Median GTV Volume (cm^3^)	0.48
		Median BED_10_ (Gy)	81.6
		Median EQD_2_ (Gy)	68
		Median TT (%)	83.5
		SRS2	
		Single fraction	19 (59)
		Fractionated	13 (41)
		Median GTV volume (cm^3^)	1.35
		Median BED_10_ (Gy)	65.1
		Median EQD_2_ (Gy)	54.3
		Median TT (%)	84
		Median interval SRS1 to SRS2 (months)	9.7
		Surgical resection prior to SRS2	9 (28)

*WBRT, whole brain radiation therapy; KPS, Karnofsky Performance Scale; GTV, gross tumor volume*.

Of the 32 lesions, 24 were associated with controlled extracranial disease and 4 with no extracranial disease during SRS1. A majority of the lesions treated during SRS1 were in patients with favorable DS-GPA, with 93% having a DS-GPA of 2–4. At SRS1, 26 (81%) were treated with SRS alone and 6 (19%) lesions received post-operative SRS to tumor cavity. The median volume for the GTV was 0.48 cm^3^ (range, 0.02–6.70 cm^3^). The median total dose at SRS1 was 24 Gy (range, 18–30 Gy). Thirty (94%) lesions were treated with a single fraction (range, 18–28 Gy) and 2 (6%) lesions received fractionated SRS (1 lesion was treated to 30 Gy in 3 fractions and another was treated to 24 Gy in 3 fractions). Due to different fractionated schemes used, the biologically effective dose (BED) and equivalent dose in 2 Gy fractions (EQD_2_) for direct comparison were calculated for an α/β ratio of 10. The formula used to calculate BED was ${\text{BED}}_{\alpha /\beta }=N\times d\times \left[1+\frac{d}{\alpha /\beta }\right]\;$, and the formula used to calculate EQD_2_ was ${\text{EQD}}_{2}=N\times d\times \frac{d+\alpha /\beta }{2+\alpha /\beta }$, with *N* = number of fractions, *d* = dose, α = linear coefficient reflecting cellular radiosensitivity, and β = quadratic coefficient reflecting cell repair mechanisms. The median BED was 81.6 Gy (range, 43.2–106.4 Gy). The median EQD_2_ was 68 Gy (range, 36.0–88.7 Gy). The median prescribed isodose line was 83.5% (range, 69–96%).

The median time to repeat SRS was 9.7 months (range, 2.5–56.9 months). During SRS2, 18 lesions were associated with controlled extracranial disease and 5 with no extracranial disease. A majority of the lesions treated during SRS2 were also in patients with favorable DS-GPA, with 87% having a DS-GPA of 2–4. Prior to SRS2, nine (28%) lesions had an interval resection of recurrent brain metastasis and received post-operative repeat SRS to tumor cavity. The median volume for GTV was 1.35 cm^3^ (range, 0.11–34.93 cm^3^). The median total dose was 26.5 Gy (range, 18–36 Gy). 19 (59%) lesions were treated with a single fraction (range, 18–27 Gy) and 13 (41%) lesions received fractionated SRS (most commonly 6 Gy × 5 and 10 Gy × 3). The median BED was 65.1 Gy (range, 42.6–99.9 Gy). The median EQD_2_ was 54.3 Gy (range, 35.5–83.3 Gy). The median prescribed isodose line was 84% (range, 69–93%).

Fifteen (47%) of the lesions were irradiated within 6 months of receiving target therapy. These included immunotherapies, kinase inhibitors, and monoclonal antibodies (Table 2).

**Table 2:** Targeted therapies used in 15 lesions within 6 months of SRS1 or SRS2.

Systemic agent	Value (%)
Sunitinib	3 (20)
Sorafenib	2 (13)
Dabrafenib	2 (13)
Trastuzumab	2 (13)
IL-2	2 (13)
Pembrolizumab	2 (13)
Ipilimumab	1 (7)
Erlotinib	1 (7)

*SRS, stereotactic radiosurgery*.

### Overall Survival

**Figure 1:**
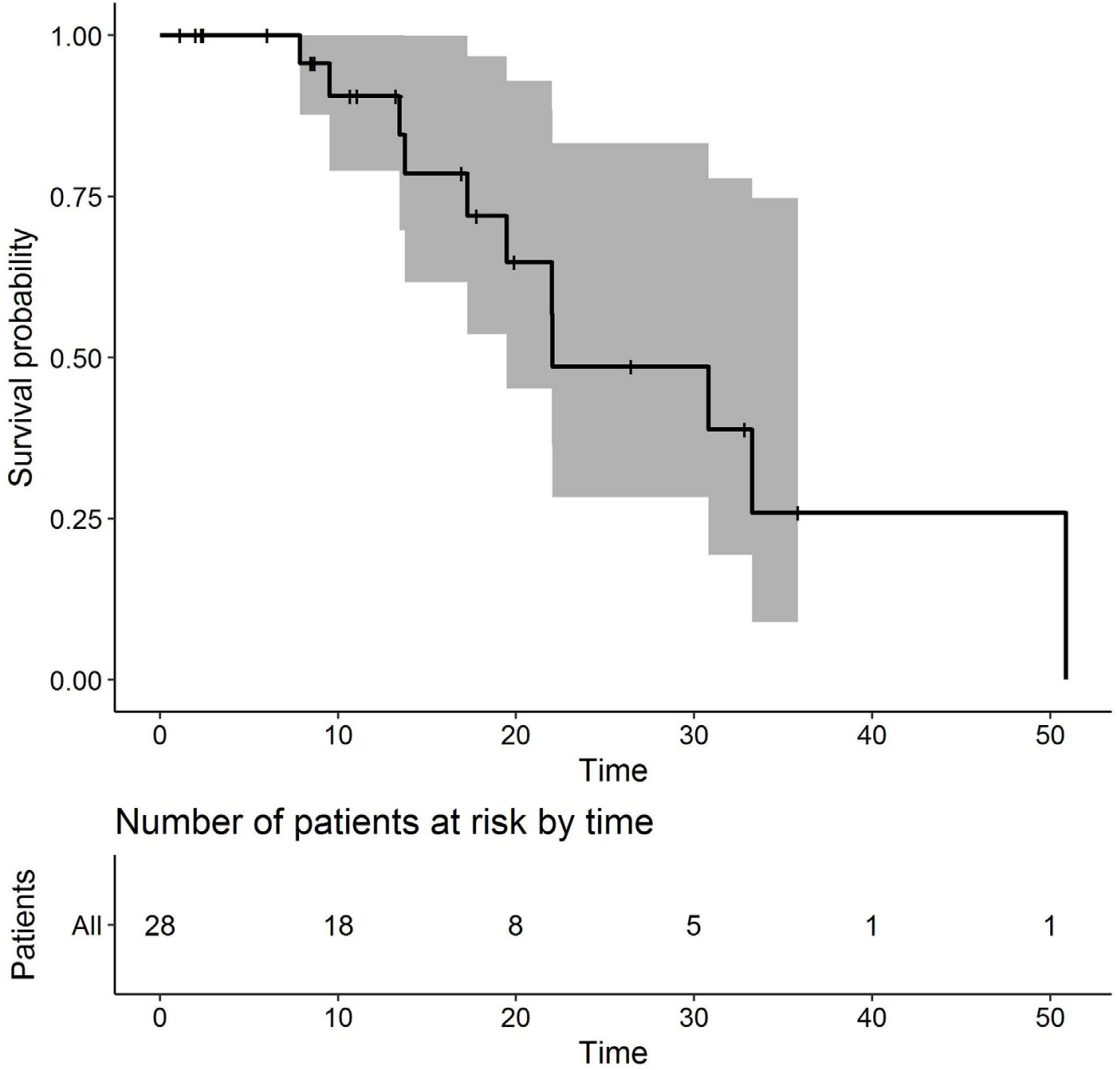
Kaplan–Meier plot of overall survival (months).

Median OS time was estimated as 22.0 months. Survival at 12 months was 90.6% (95% CI 79.0–100%, Figure 1). Survival at 18 months was 72.0% (95% CI 53.6–96.7%). Survival at 24 months was 48.6% (95% CI 28.4–83.3%).

### Local Control

**Figure 2:**
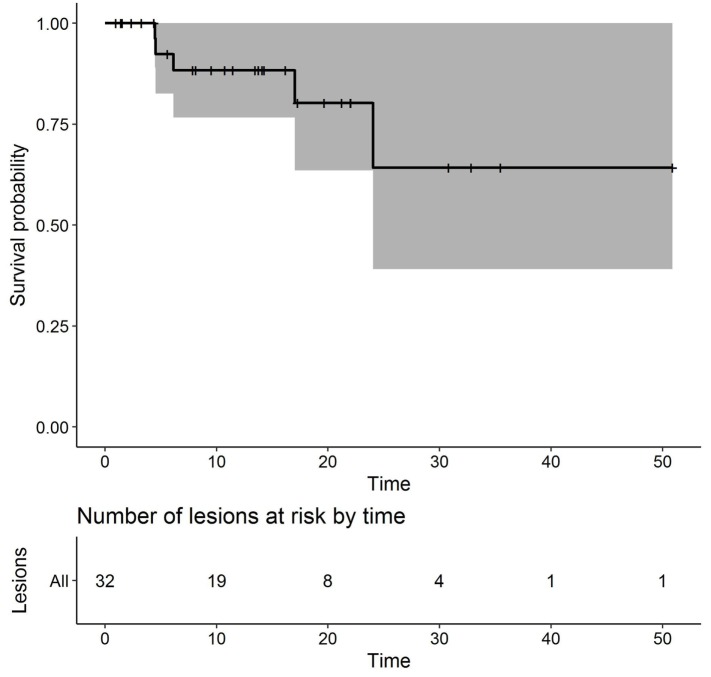
Kaplan–Meier plot of local failure-free survival (months).

Median LFFS time after SRS2 was estimated as 13.6 months. Five lesions recurred locally after SRS2. The overall local control rate following SRS2 was 84.4%. The 1- and 2-year local control rates are 88.3% (95% CI, 76.7–100%) and 80.3% (95% CI, 63.5–100%), respectively (Figure 2). The median time to local failure after SRS2 was 6.1 months (range, 4.5–24 months). Salvage therapy consisted of surgical resection for two lesions, repeat SRS for two lesions, and WBRT for one lesion that was also associated with leptomeningeal spread.

On univariate analysis, histology, prior WBRT, resection prior to SRS2, EQD2 at SRS1 or SRS2, prescribed isodose line at SRS1 or SRS2, time interval between SRS1 and SRS2, GTV volume at SRS1 or SRS2, single versus multiple fractions at SRS1 or SRS2, and use of concurrent systemic therapy with SRS1 or SRS2 were not significantly associated with local failure.

### Radionecrosis

Six lesions developed radionecrosis after SRS2. The overall rate of radionecrosis following SRS2 was 18.8%. The median time to radionecrosis after SRS2 was 3.9 months (range, 2.2–15.4 months). All lesions that developed radionecrosis received single fraction SRS for both SRS1 and SRS2. One patient who developed radionecrosis after SRS2 received prior WBRT.

Treatment for radionecrosis consisted of conservative management alone with steroids for five lesions, and surgical resection for one lesion.

**Figure 3:**
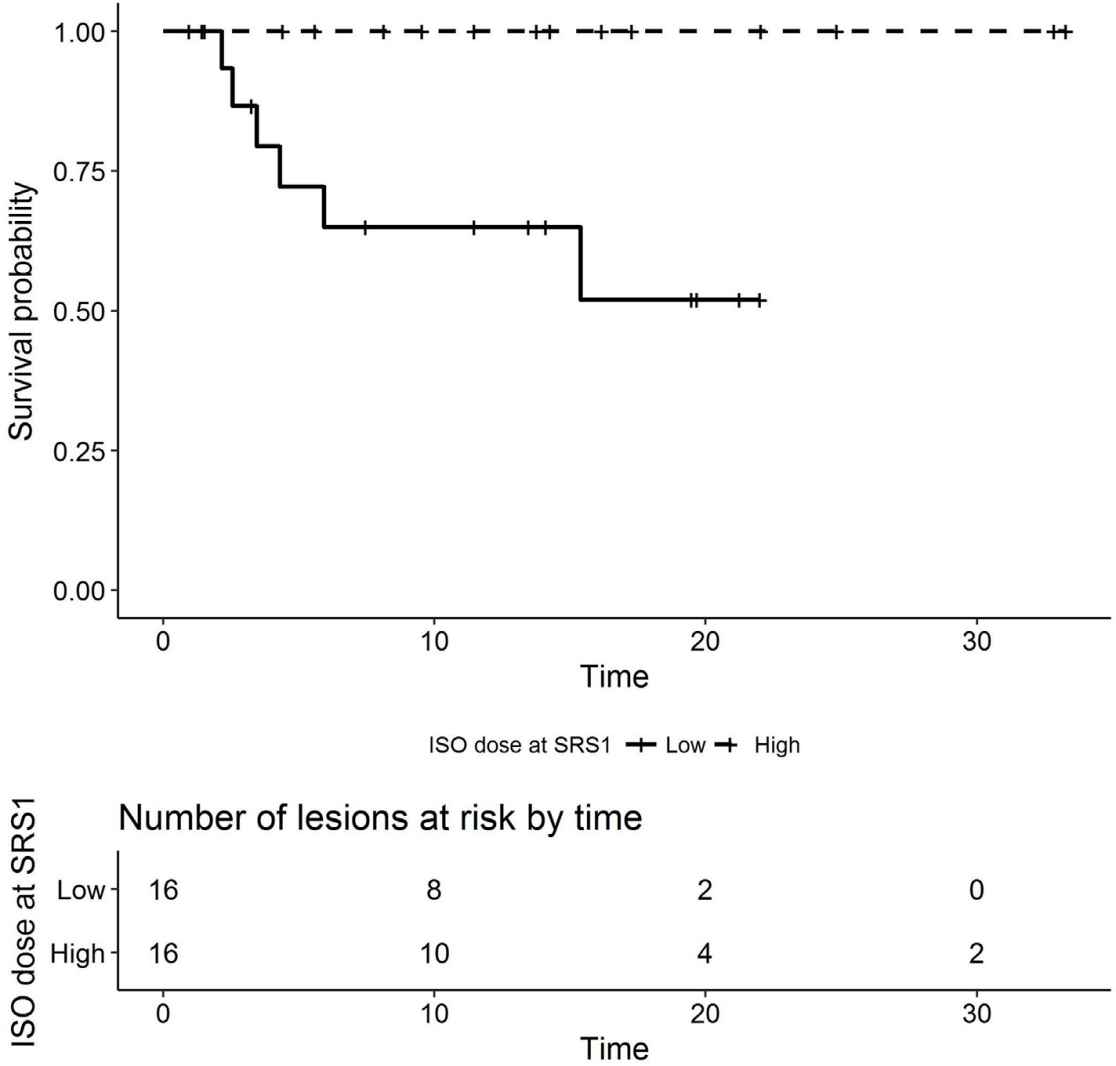
Kaplan–Meier plot of radionecrosis-free survival by prescribed isodose line at SRS1 (months, low versus high dichotomized by median dose 83.5%). SRS, stereotactic radiosurgery.

**Figure 4:**
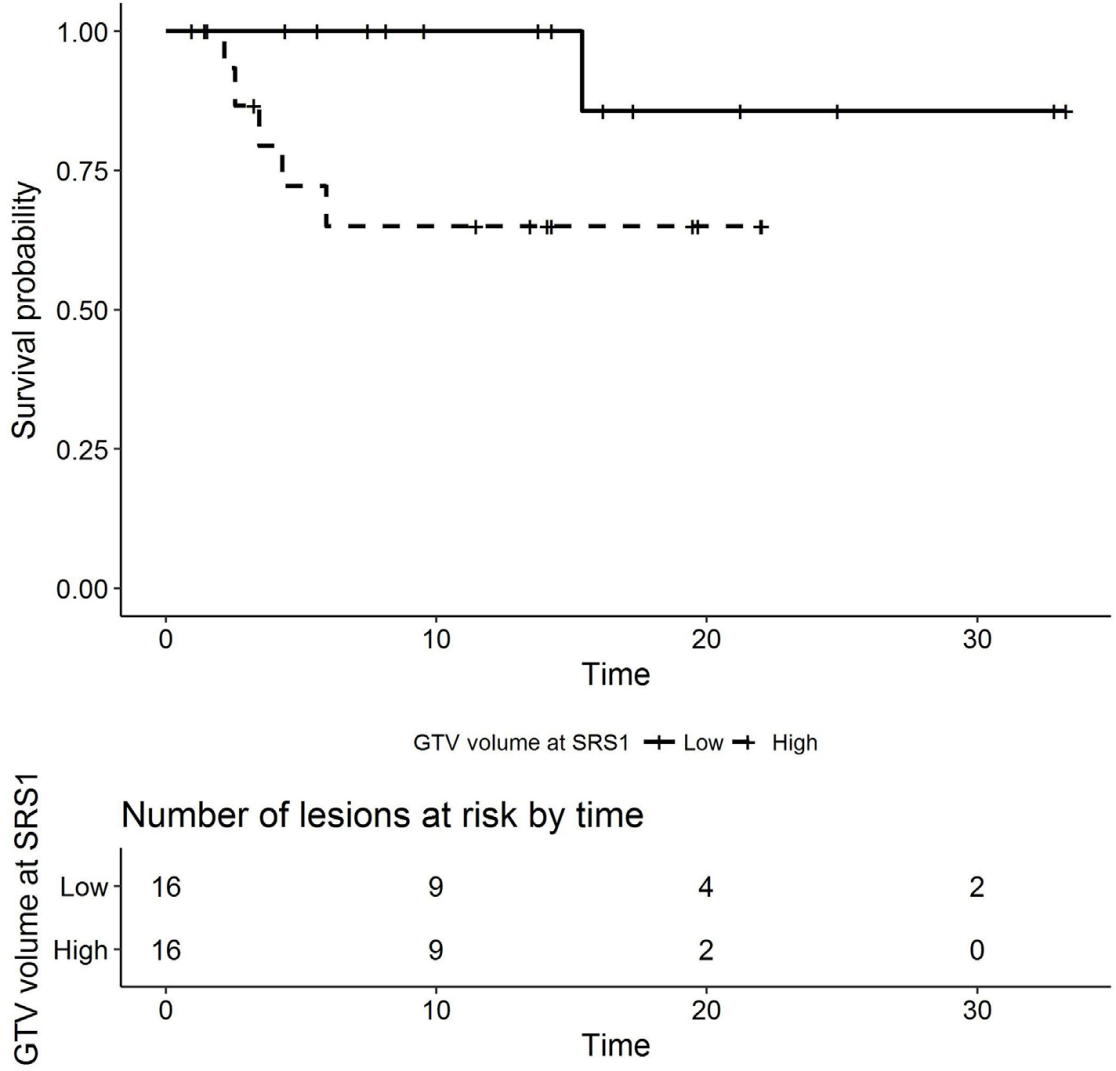
Kaplan–Meier plot of radionecrosis-free survival by GTV volume at SRS1 (months, low versus high dichotomized by median volume 0.4825). SRS, stereotactic radiosurgery; GTV, gross tumor volume.

In the univariate analyses, higher prescribed isodose line at SRS1 was associated with a lower hazard of radionecrosis (HR 0.886, 95% CI 0.788–0.995, *p* = 0.033, Figure 3). In addition, higher GTV volume at SRS1 was associated with higher HR (1.55, 95% CI 1.05–2.29, *p* = 0.015, Figure 4). Histology, prior WBRT, resection prior to SRS2, EQD2 at SRS1 or SRS2, prescribed isodose line at SRS2, time interval between SRS1 and SRS2, GTV volume at SRS1 or SRS2, single versus multiple fractions at SRS1 or SRS2, and use of concurrent systemic therapy with SRS1 or SRS2 were not observed significantly associated with radionecrosis.

The median GTV volume at SRS1 for lesions with radionecrosis was 2.71 cm^3^ compared to 0.32 cm^3^ for lesions without radionecrosis. At SRS1, 100% of the lesions with radionecrosis received single fractionated treatment, compared to 92% of lesions without radionecrosis. The median BED/EDQ2 at SRS1 for lesions with radionecrosis were 70.8/59 Gy compared to 81.6/68 Gy at for lesions without radionecrosis. The median prescribed isodose line at SRS1 for lesions with radionecrosis was 80% compared to 87.5% for lesions without radionecrosis. 33% of lesions with radionecrosis received concurrent systemic therapy with SRS1 compared to 19.2% of lesions without radionecrosis.

The median time interval between SRS1 and SRS2 was 12.1 months for those that developed radionecrosis and 9.5 months in those that did not. Prior to SRS2, 16.7% of lesions with radionecrosis underwent interval surgical resection after SRS1, compared to 30.8% of the lesions without radionecrosis. The median GTV volume at SRS2 for lesions with radionecrosis was 1.03 cm^3^ compared to 1.81 cm^3^ for lesions without radionecrosis. At SRS2, 100% of lesions with radionecrosis received single fractionated treatment, compared to 50% of the lesions without radionecrosis. The median BED/EQD2 at SRS2 for lesions with radionecrosis was 81.6/68 Gy compared to 60/50 Gy for lesions without radionecrosis. The median prescribed isodose line at SRS2 for lesions with radionecrosis was 82% compared to 84% for lesions without radionecrosis. 50% of lesions with radionecrosis received concurrent systemic therapy with SRS2 compared to 26.9% of lesions without radionecrosis.

## Discussion

The optimal treatment strategy for recurrent brain metastases previously treated with SRS remains uncertain. Possible treatment options include WBRT, surgical resection with or without adjuvant SRS, SRS alone, or close observation. The routine use of WBRT in the upfront management of brain metastases has fallen out of favor because of long-term neurotoxicity and detriments to quality of life (7, 16–19). Similarly, these risks exist in the salvage setting. WBRT or best supportive care is often utilized in patients with a limited life expectancy, poor performance status, uncontrolled systemic disease, or a high burden of intracranial disease. WBRT is less ideal in patients with limited brain metastases and a favorable prognosis. For this population, alternative treatment options include surgical resection or repeat SRS.

The advantages of surgical resection include pathological confirmation of tumor recurrence versus radionecrosis and quick palliation of symptoms secondary to mass effect. However, salvage surgery may require adjuvant radiation to decrease the risk of local recurrence (20), can be morbid (21, 22), and may be limited by performance status and disease location.

While the data are sparse, repeat SRS is another option for patients who are not ideal candidates for salvage WBRT or surgery. McKay et al. demonstrated 1-year local control rate of 79%, 1-year OS rate of 70% (median survival not reached at time of analysis), and a 24% risk of developing radionecrosis using Gamma Knife for repeat SRS (23). Koffer et al. demonstrated 1-year local control rate of 61%, median survival of 8.8 months (1-year OS rate of 37.5%), and a 16.7% risk of developing radionecrosis, also using Gamma Knife for repeat SRS (24). Minniti et al. delivered fractionated repeat SRS and demonstrated 1-year local control rate of 70%, median survival of 10 months (1-year OS rate of 37%), and a 19% risk of developing radiologic changes suggestive of radionecrosis (25). Our study showed a 1-year local control rate of 88.3%, median survival of 22 months (1-year OS rate of 90.6%), and radionecrosis rate of 18.8%. Across these studies, 1-year local control rates ranged between 61 and 88% with radionecrosis risk of 16 and 25%. We suspect that the higher OS seen in our series is due to selection bias and tumor type. For instance, Koffer et al. included a large proportion of individuals with NSCLC and SCLC, while our series included a large portion of individuals with renal cell carcinoma and melanoma, where differing immunotherapies and molecular targets are emerging.

The use of LINAC-based SRS may allow for strategies that can reduce the risk of radionecrosis compared to Gamma Knife in the retreatment setting. Such strategies include fractionated treatment schedules allowing for normal tissue repair (26, 27) and treating to a higher isodose line, thus reducing the maximum dose within the target. Estimating survival outcomes remains a challenge because of limited patient numbers, selection bias, and variable disease characteristics.

As discussed earlier, the rate of radionecrosis following SRS2 in our series and others approached 20%. On univariate analysis, higher prescribed isodose line (*p* = 0.033) and higher GTV volume (*p* = 0.015) at SRS1 were associated with radionecrosis. Although not statistically significant, there was a trend toward lower risk of radionecrosis with interval surgical resection, fractionated SRS, lower total EQD_2_ (<50 Gy), and lack of concurrent systemic therapy at SRS2.

The interpretation of our results is limited by the small patient numbers and single-institutional retrospective study design. There are multiple biases in collecting these data, which may have led to an underestimation of the risk of radionecrosis or overestimation of survival by preferentially selecting younger individuals with a low burden of systemic disease and limited burden of intracranial disease for repeat SRS. It is possible that some patients with radionecrosis were not identified because of loss to follow-up. Multiple dose and fractionation schedules were used, which limit our ability to identify specific variables that might contribute to local control and development of radionecrosis. Prospective data and standard treatment protocols are required for future study.

Despite these limits, we believe this study contributes to the limited data on repeat SRS for in-field recurrence and offers additional insights into this increasingly encountered clinical scenario. In the appropriate setting, repeat LINAC-based SRS to the same isocenter is a reasonable approach for patients with recurrent disease who wish to avoid surgical resection or WBRT with excellent local control and an acceptable risk of radionecrosis. Future studies are required to better define variables such as total EQD2 and role of fractionation that impact disease and patient-related outcomes.

## Ethics Statement

This retrospective review has been approved by the Vanderbilt University Medical Center Internal Review Board.

## Author Contributions

NR and MS contributed to the writing, design, organization, data gathering, editing, and submission of this manuscript. PP, RC, and GL contributed to data gathering. ZZ contributed to statistical analysis. LC, AC, and AA contributed to discussion on repeat SRS.

## Conflict of Interest Statement

The authors declare that the research was conducted in the absence of any commercial or financial relationships that could be construed as a potential conflict of interest.
